# Supplementary material for: Perilla frutescens Extracts Enhance DNA Repair Response in UVB Damaged HaCaT Cells
Source: Nutrients. 2021 Apr 12;13(4):1263. doi: 10.3390/nu13041263 (PMC8070160; doi:10.3390/nu13041263)
Supplement: Supplementary file 1 [file nutrients-13-01263-s001.pdf]

Supplementary Figures

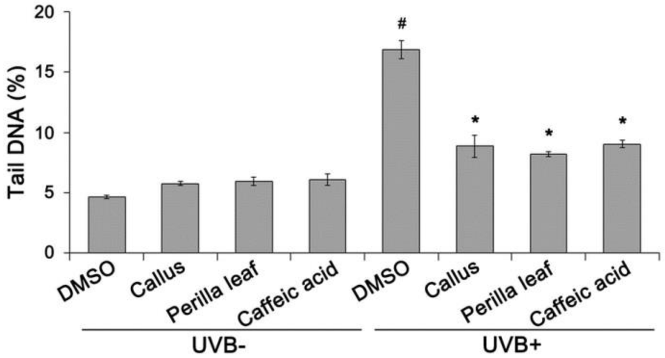

Figure S1. DNA damage was assessed using the Comet assay. Tail DNA percentages were determined. Comet images revealed different degrees of DNA damage. HaCaT cells were cultured in 6-well plates and pretreated with Perilla leaf or callus extracts (at 0.1 μg/ml), caffeic acid (150 μM; the positive control), or DMSO (the negative control) for 12 hours, and then exposed to UVB (30 mJ/cm2). Image fluorescence intensities were obtained using the Comet assay. . #p<0.05; significant versus UVB non-treated and DMSO controls. *p<0.05; significant versus UVB treated and DMSO controls.

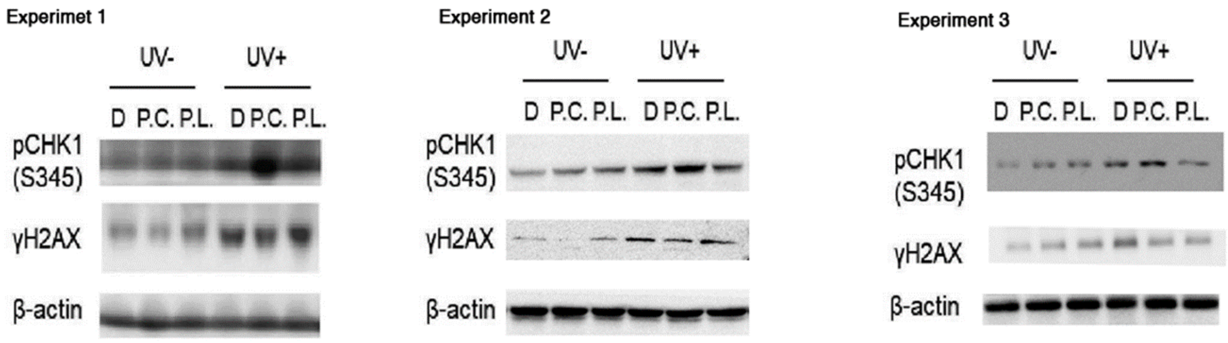

Figure S2. All images of western blot for pCHK1 (S345) and γH2AX proteins in HaCaT cells treated with Perilla leaf or callus extracts at 0.1ug/ml (D: DMSO treatment; P.C; Perilla callus; P.L: Perilla leaf). β-Actin was used as the loading control. The experiments were performed in three biological independent trials. Keratinocytes were treated with Perilla extracts overnight, washed with PBS, and irradiated with UVB (30 mJ/cm2), and 3 hrs later, pellets were harvested for western blotting.
